# Micheliolide ameliorates renal fibrosis by suppressing the Mtdh/BMP/MAPK pathway

**DOI:** 10.1038/s41374-019-0245-6

**Published:** 2019-04-11

**Authors:** Fenfen Peng, Hongyu Li, Shuting Li, Yuxian Wang, Wenting Liu, Wangqiu Gong, Bohui Yin, Sijia Chen, Ying Zhang, Congwei Luo, Weidong Zhou, Yihua Chen, Peilin Li, Qianyin Huang, Zhaozhong Xu, Haibo Long

**Affiliations:** 1 Department of Nephrology, Zhujiang Hospital, Southern Medical University, Guangzhou, 510280 China; 2 Department of Gerontology, ZhuJiang Hospital, Southern Medical University, Guangzhou, 510280 China; 3 Department of Nephrology, The First Hospital of Changsha, Changsha, 410000 China; 4 Department of Nephrology, The Second Affiliated Hospital, Guangzhou Medical University, Guangzhou, 510260 China

**Keywords:** Drug regulation, End-stage renal disease

## Abstract

Micheliolide (MCL), derived from parthenolide (PTL), is known for its antioxidant and anti-inflammatory effects and has multiple roles in inflammatory diseases and tumours. To investigate its effect on renal disease, we intragastrically administrated DMAMCL, a dimethylamino Michael adduct of MCL for in vivo use, in two renal fibrosis models–the unilateral ureteral occlusion (UUO) model and an ischaemia-reperfusion injury (IRI) model and used MCL in combination with transforming growth factor beta 1 (TGF-β1) on mouse tubular epithelial cells (mTEC) in vitro. The expression of fibrotic markers (fibronectin and α-SMA) was remarkably reduced, while the expression of the epithelial marker E-cadherin was restored after DMAMCL treatment both in the UUO and IRI mice. MCL function in TGF-β1-induced epithelial-mesenchymal transition (EMT) in mTEC was consistent with the in vivo results. Metadherin (Mtdh) was activated in the fibrotic condition, suggesting that it might be involved in fibrogenesis. Interestingly, we found that while Mtdh was upregulated in the fibrotic condition, DMAMCL/MCL could suppress its expression. The overexpression of Mtdh exerted a pro-fibrotic effect by modulating the BMP/MAPK pathway in mTECs, and MCL could specifically reverse this effect. In conclusion, DMAMCL/MCL treatment represents a novel and effective therapy for renal fibrosis by suppressing the Mtdh/BMP/MAPK pathway.

## Abstract (web summary)

Micheliolide (MCL) is known for its antioxidant and anti-inflammatory effects and has multiple effects in inflammatory diseases and tumors. The authors studied the effects of an MCL derivative in two renal fibrosis models. They found that the metastasis adhesion protein metadherin (Mtdh) was activated under fibrotic conditions and that MCL suppressed its expression via the BMP/MAPK pathway.

## Introduction

Micheliolide (MCL) and parthenolide (PTL) are compounds isolated from the herbs *Michelia compressa* and *Michelia champaca* [1]. MCL, as a guaianolide sesquiterpene lactone derivative of PTL, is more stable and soluble in plasma and exhibits promising therapeutic efficacy towards inflammation and cancer [2, 3]. The underlying mechanism might be attributed to its specific structure, α-methylene-γ-lactone, which allows MCL to alkylate p65 cysteine-38 and inhibit the binding of the p65/NF-κB subunit to DNA, exerting an NF-κB antagonist effect [4]. The dimethylamino Michael adduct of MCL (DMAMCL), a water-soluble pro-drug of MCL typically used in vivo, slowly and continuously releases MCL into plasma, increasing its oral bioavailability and enhancing its therapeutic potential [1, 5, 6]. In recent years, a wealth of studies have identified MCL efficacy on inflammatory diseases and tumours, including rheumatoid arthritis, acute myelogenous leukaemia, LPS-induced neuroinflammatory responses, inflammatory bowel diseases and glioma in animal models [1–3, 6, 7]. However, there is no evidence regarding MCL function in fibrotic diseases to date.

Our previous studies on MCL showed its anti-inflammatory effect on adipohepatic damage in diabetic mice [8] and the alleviation of advanced oxidation protein product (AOPP)-induced injury on mesangial cells and podocytes [9]. Therefore, MCL appears to exert an effect on kidney diseases. We questioned whether MCL also has a therapeutic effect on renal fibrosis.

Renal fibrosis is the most common feature of chronic kidney diseases (CKD) progressing to end-stage renal disease. This process involves the loss of the renal parenchyma and the excessive accumulation of extracellular matrix (ECM) [10, 11]. Renal fibrosis exists in all processing forms of CKD, irrespective of the initial injury [12]. Located at the epicentre and being vulnerable to direct or secondary tubular damage, tubular epithelial cells (TECs) are thought to be an early responder to drive the inflammatory and fibrotic process [13–15]. Fibrosis is difficult to reverse or cure. Researchers have attempted to block the critical pro-fibrotic cytokine TGF-β1 with neutralizing antibodies, but the approach failed to show satisfactory efficacy in clinical trials [16–18]. Determining the underlying mechanism in renal fibrogenesis and developing an effective remedy to maintain renal function are still topics of concern for researchers and society.

Metadherin (Mtdh), also known as lysine-rich CEACAM1 co-isolated (LYRIC)/3D3 and astrocyte elevated gene-1 (AEG-1), was initially discovered as an oncogene that is upregulated in various types of cancers, such as breast carcinoma and hepatoma [19, 20]. Studies focusing on Mtdh to date have shown that it is a multi-functional factor involved in many important processes, including cell proliferation, apoptosis, autophagy, migration, invasion, metastasis and chemotherapy resistance, via activating signalling pathways such as NF-κB, PI3K/AKT and MAPK [19, 21–24]. Epithelial-mesenchymal transition (EMT) is a process through which Mtdh participates in tumourigenesis [25, 26]; it is also known as an important mechanism in fibrosis. However, no study has yet shown a relationship between Mtdh and fibrosis.

Mtdh is expressed in not only the tumour condition but also in healthy organs, and the kidney is one such organ with high Mtdh expression [20]. According to our previous study, Mtdh is upregulated in podocytes during hyperglycaemia (HG) and modulates podocyte apoptosis by binding to miR-30 s [27]. Evidence supports a role for Mtdh in kidney diseases, including renal fibrogenesis.

In this study, we highlight the therapeutic efficacy of MCL on renal fibrosis in vivo and in vitro and further investigate the underlying mechanism of Mtdh as a target of MCL in this condition.

## Materials and methods

### MCL and DMAMCL

MCL and DMAMCL were synthesized by Accendatech Co., Ltd. (Tianjin, China). DMAMCL was freshly dissolved in normal saline (NS) daily for the animal experiments. MCL was prepared in dimethyl sulphoxide (DMSO, Sigma-Aldrich, Missouri, USA) at a concentration of 20 mM and stored at −20 °C.

### Cytotoxicity assay

An MTT assay was used to detect the cytotoxicity of MCL towards mouse tubular epithelial cells (mTECs, a gift from Dr Jeffrey B. Kopp, NIH, Bethesda, MD, USA). A total of 1 × 10^4^ cells/ml was seeded in 96-well plates and cultured in a 37 °C incubator with 5% CO_2_. Upon reaching 70% confluence, the cells were treated with MCL (0, 1.25, 2.5, 5, or 10 μM) for 24 h. Then, 5 mg/ml 3-(4,5-dimethylthiazol-2-yl)-2,5-diphenyltetrazolium bromide (MTT, Sigma-Aldrich, St. Louis, MO, USA) was added to the media (10 μl/well) for 4 h. The supernatant was carefully aspirated, and 150 μl/well of DMSO, was added to the medium to dissolve the remaining crystals. After low-speed vibration for 10 min at room temperature, the optical density of all wells was measured at 490 nm using a microplate Reader (SpectraMax® M5, Molecular Devices, California, USA). The MTT assay was repeated independently at least 3 times, and the viability curve of mTECs was generated using GraphPad Prism 6 software (California, USA).

### Animal model

For the UUO model, C57BL/6 mice (males, 8 weeks old, weighing 18–22 g) were purchased from and housed at the Southern Medical University Laboratory Animal Centre (Guangzhou, China). The mice were divided into three groups as follows (*n* = 6 mice per group): (1) sham-operated mice with a normal saline (NS) gavage (sham group); (2) unilateral ureteral occlusion (UUO) mice with an NS gavage (vehicle group); and (3) UUO mice with a DMAMCL gavage (DMAMCL group, 25 mg/kg•d). The UUO model was generated using an established procedure, as previously described [28]. Following daily treatment with NS/DMAMCL by intragastric administration, the mice were sacrificed on day 14, and the renal tissue was collected under appropriate conditions.

For the IRI model, C57BL/6 mice (males, 8 weeks old, weighing 18–22 g) were purchased from Pengyue Laboratory Animal Breeding Company (Shandong, China) and housed at the Southern Medical University Laboratory Animal Center (Guangzhou, China). The mice were divided into three groups as follows (*n* = 6 mice per group): (1) sham-operated mice with a normal saline (NS) gavage (sham group); (2) IRI mice with an NS gavage (vehicle group); and (3) IRI mice with a DMAMCL gavage (DMAMCL group, 25 mg/kg•d). The IRI surgery was conducted using an established procedure [28]. Drug treatment was started from the day of surgery and lasted 11 days. On the 10th day, the mice were underwent unephretomy of the contralateral kidney.

For these two animal experiments, the mice were raised in a specific pathogen-free environment with a fixed temperature (23 ± 3 °C), constant humidity (55 ± 15%) and a 12-hour light-dark cycle, and they were provided standard food and water. The animal experimental protocols were approved by the Southern Medical University Ethics Committee (Approval No. L2015117) and strictly complied with ethical principles throughout the whole experiment.

### Renal tissue pathology

Kidney tissues were fixed with a 4% paraformaldehyde solution, embedded in paraffin, and sectioned into 4-μm-thick slices. The kidney tissue sections were stained with Masson’s trichrome dyes and then examined under a light microscope.

### Immunohistochemical staining

Immunohistochemical staining was performed as previously described, with a slight modification [29]. A GTVision^TM^ III Detection System/Mo & Rb (Gene Tech, Shanghai, China) was used following the manufacturer’s instructions. All antibodies were freshly diluted with 2% goat serum (Boster, Wuhan, China). The LYRIC (Mtdh) antibody (Abcam, Cambridge, Britain) was used at a 1:100 dilution, the fibronectin antibody (BD Biosciences, USA) was used at a 1:200 dilution, the α-SMA antibody (Sigma-Aldrich, Missouri, USA) was used at a 1:250 dilution and the E-cadherin antibody (BD Biosciences, California, USA) was used at a 1:100 dilution. Slides were counterstained with haematoxylin followed by a bluing reagent. Negative controls were treated with PBS instead of primary antibodies.

### Cell culture

The mTECs were cultured in DMEM/F12 (HyClone, Utah, USA) supplemented with 5% FBS (Gibco, Massachusetts, USA) in a humidified incubator with a 5% CO_2_ at 37 °C. Upon reaching approximately 70% confluence, the cells were serum-starved for 12 h and then treated with recombinant human TGF-β1 (R&D Systems, Minnesota, USA) for the time course or co-incubated with MCL (0, 1.25, 2.5, 5, or 10 μM), as indicated.

### Mtdh stable cell line construction

The mTECs were obtained to construct an Mtdh stable overexpression cell line in this study. mTECs were seeded in 12-well plates and cultured as described above before various concentrations of puromycin were added (0–40 μg/ml). The viable count of mTECs was calculated, and the minimum concentration of puromycin that caused complete mTECs death was recorded. The process was repeated twice, and the screening concentration of 10 ng/ml was used in subsequent experiments. Mtdh recombinant packaged lentivirus particles (LV-Mtdh) were constructed by GENECHEM (Nanjing, China), and a lentivirus vector lacking Mtdh sequences was used as a negative control (LV-NC). The related information regarding the Mtdh lentivirus is shown in the Supplementary Materials (sup1). mTECs were infected with packaged virus particles and polybrene (8 μg/ml) overnight, according to the manufacturer’s instructions. Fresh culture medium containing 10 ng/μl puromycin was added early the next morning. The infected cells continued to grow until they reached 95% confluence and were then reseeded into new 100-mm dishes at a 10–20% density and grow continuously for 2 weeks. The 5% FBS medium containing 10 ng/μl puromycin was replaced every 3 days until drug-resistant clones appeared. Ten clones were carefully picked, and the cloned cells were cultured, passaged, and finally frozen at −80 °C. A stable Mtdh-knockdown mTEC line was established as described above.

### Transient transfection

Mtdh sequences were inserted into a pcDNA3.1 vector to construct a plasmid targeting Mtdh (Mtdh vector) by GenePharma (Shanghai, China). The empty vector was used as a control (Negative control). mTECs were seeded into 6-well plates. Upon reaching 70% confluence, Lipofectamine^TM^ 2000 (Invitrogen, Massachusetts, USA) and a certain volume of plasmid was added to the culture medium. 6 h later, the transfection reagents were removed, fresh complete medium supplemented with 5% FBS ± MCL (10 μM) was added, and the cells were cultured for an additional24 h before harvest.

### Western blot analyses

Cell or the whole kidney tissue lysates were extracted in SDS lysis buffer containing 1 × PMSF (Aidlab, Beijing, China) and 1 × phosphatase inhibitor (Aidlab, Beijing, China). Western blots were performed as indicated with the following primary antibodies: Mtdh, fibronectin, BMPR1A, and Smad1/5/9 (Abcam, Cambridge, Britain); Col I (Boster, Wuhan, China); α-SMA (Sigma-Aldrich, Missouri, USA); E-cadherin (BD Biosciences, California, USA); p-P38 MAPK, P38 MAPK, p-ERK, ERK, and p-Smad1/5/9 (Cell Signalling Technology, Massachusetts, USA), and GAPDH (EarthOx Life Science, California, USA). The blots were subsequently incubated with an HRP-conjugated secondary antibody (EarthOx Life Science, California, USA) for 1–2 h at ambient temperature. Then, the membranes with immobilized antibodies were detected by enhanced chemiluminescence (Immobilon^TM^ Western, Millipore, Massachusetts, USA). Quantification was performed by measuring the grey scale intensity of the bands using Photoshop CS5 software (Adobe System Inc., California, USA).

### Statistical analyses

Statistical analyses were performed with SPSS 20.0 (IBM, New York, USA). All data were from at least three independent experiments and presented as the mean ± standard error of the mean (SEM). Student’s t-test was used to analyse the differences between two groups, whereas ANOVA was used for the comparisons in multiple groups, followed by Student’s *t*-test for the determination of differences between every two groups; Pairwise comparison in unequal variables was analysed by the Student-Newman-Keuls procedure or Dunnett’s T3 procedure. A *p* value < 0.05 was considered statistically significant.

## Results

### Chemical structures and toxicity of MCL and DMAMCL

**Fig. 1:**
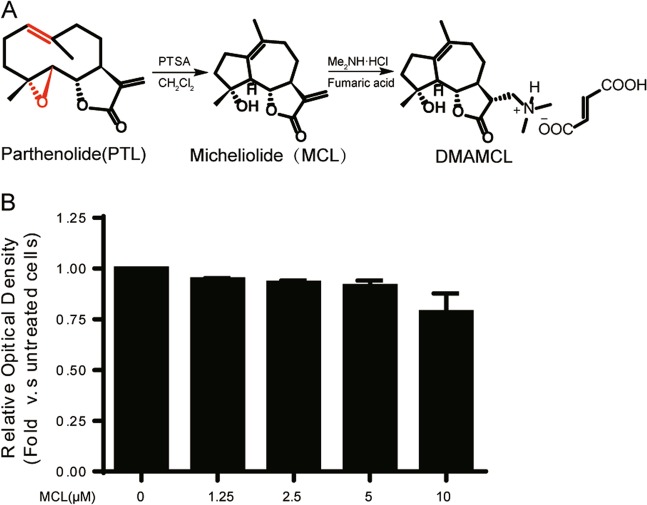
Chemical structures and cytotoxicity of DMAMCL/MCL. **a** Chemical structures of MCL and DMAMCL. **b** Evaluation of the cytotoxic effects of MCL on the viability of mTECs. mTECs were incubated with different concentrations of MCL for 24 h, and cell viability was determined using an MTT assay. The data are presented as the mean ± SEM of at least three independent experiments

MCL and DMAMCL (Fig. 1a) were synthesized as previously described [2]. We determined the effects of different concentrations of MCL (0, 1.25, 2.5, 5, 10 and 20 μM) on mTECs using an MTT assay to evaluate the cytotoxicity of MCL in vitro. After 24 h of treatment, the cell viability of mTECs cultured with 0, 1.25, 2.5, 5, and 10 μM MCL was greater over 90% (Fig. 1b), indicating that MCL does not induce significant cell toxicity at concentrations less than 10 μM in mTECs. MCL was administered at a concentration of 10 μM in subsequent experiments.

### DMAMCL ameliorates renal fibrosis in a UUO model

**Fig. 2:**
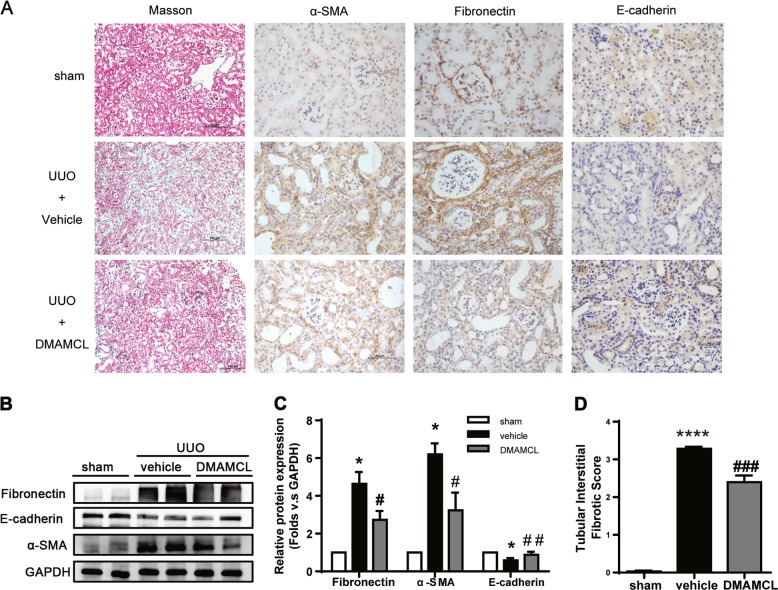
DMAMCL attenuates renal fibrosis in UUO mice. **a** Representative micrographs of Masson’s trichrome staining and IHC staining for α-SMA, fibronectin, and E-cadherin in the obstructed kidneys. Scale bar in Masson’s trichrome staining is 100 μm; in IHC staining is 50 μm. **b** Representative bands from Western blot analyses of the levels of the α-SMA, fibronectin, and E-cadherin proteins in kidney tissues from the UUO mice. **c** Relative protein levels as determined by the Western blot assay (**b**). *P < 0.05 compared with the sham group; #*P* < 0.05 compared with the vehicle group; ##*P* < 0.01 compared with the vehicle group (*n* = 6 mice per group). **d** Quantification of renal tubular interstitial fibrotic score. ****P < 0.0001 compared with the sham group; ###*P* < 0.001 compared with the vehicle group (*n* = 6 mice per group). The data are presented as the mean ± SEM of at least three independent experiments

We initially investigated the effect of DMAMCL, the pro-drug of MCL, on renal fibrosis in a mouse UUO model. DMAMCL was administered the day after the operation via gavage at a dosage of 25 mg/kg per day (*n* = 6 animals per group), and NS was used as a vehicle control. As shown in Fig. 2a, UUO mice exhibited remarkable interstitial inflammation and collagen deposition in renal tissue stained with Masson’s trichrome dye. Fibronectin and α-SMA expression were upregulated, while E-cadherin expression was downregulated. The DMAMCL intervention inhibited the upregulation of α-SMA and fibronectin expression and restored E-cadherin expression in UUO mice (Fig. 2b, c); inflammatory cell infiltration and interstitial fibrosis scores (Fig. 2d) were significantly reduced. The above results suggest that DMAMCL relieved renal fibrosis in the UUO model.

### DMAMCL ameliorates renal fibrosis in an IRI Model

**Fig. 3:**
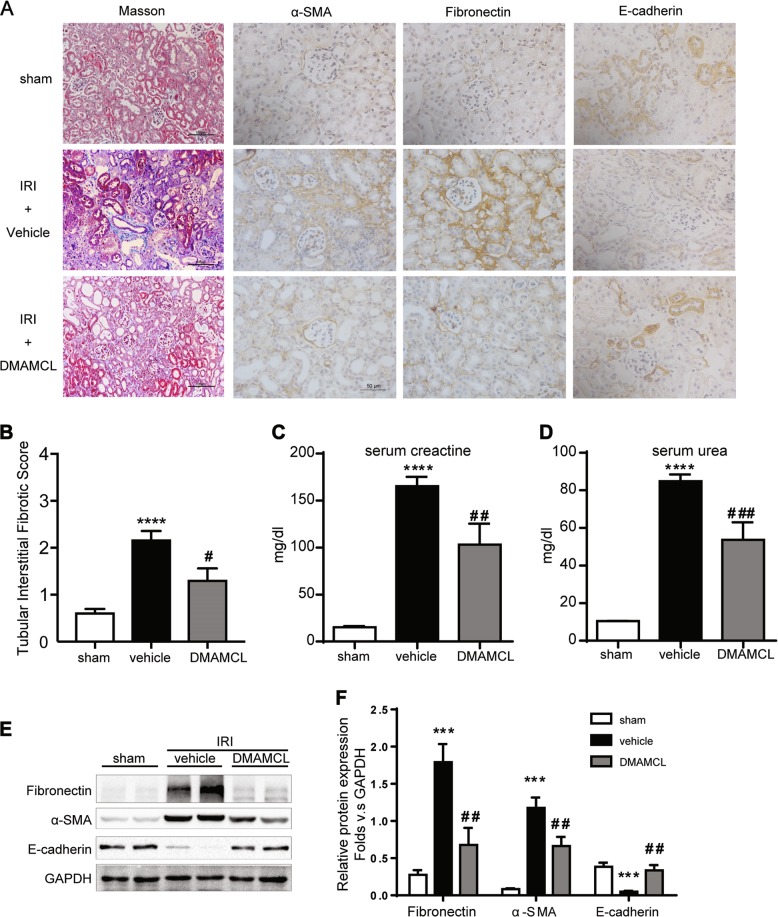
DMAMCL protects kidney from fibrosis in the IRI mice. **a** Representative micrographs of Masson’s trichrome staining and IHC staining for α-SMA, fibronectin, and E-cadherin in the injured kidneys. Scale bar in Masson’s trichrome staining is 100 μm; in IHC staining is 50 μm. **b** Quantification of renal tubular interstitial fibrotic score. ****P < 0.0001 compared with the sham group; #*P* < 0.05 compared with the vehicle group. **c** Serum creatine level in the IRI mice. ****P < 0.0001 compared with the sham group; ##*P* < 0.01 compared with the vehicle group. **d** Serum urea level in the IRI mice. ****P < 0.0001 compared with the sham group; ###*P* < 0.001 compared with the vehicle group. **e** Representative bands from Western blot analyses of the levels of the α-SMA, fibronectin, and E-cadherin proteins in kidney tissues from the IRI mice. **f** Relative protein levels as determined by the Western blot assay (**e**). ***P < 0.001 compared with the sham group; ##*P* < 0.01 compared with the vehicle group. n = 6 mice per group, all the data are presented as the mean ± SEM of at least three independent experiments

To further examine the efficacy of DMAMCL in chronic kidney diseases, the 11-day IRI model, which is a renal function detection model was conducted. Consistent with the result of UUO model, in IRI mice, DMAMCL also lessened fibrotic ECM accumulation compared with the vehicle IRI group with reduced Masson positive staining areas (Fig. 3a) and a lower fibrotic score (Fig. 3b). At the same time, renal injury was reduced, with downregulated serum creatine (Fig. 3c) and serum urea (Fig. 3d) levels. The fibrotic markers fibronection and α-SMA were significantly decreased, while E-cadherin protein was slightly restored (Fig. 3e, f). The above results indicate that DMAMCL can protect the kidney from fibrogenesis. However, the underlying mechanism remains unknown.

### DMAMCL/MCL suppresses EMT in vivo, and Mtdh might be a novel mediator in renal fibrosis

**Fig. 4:**
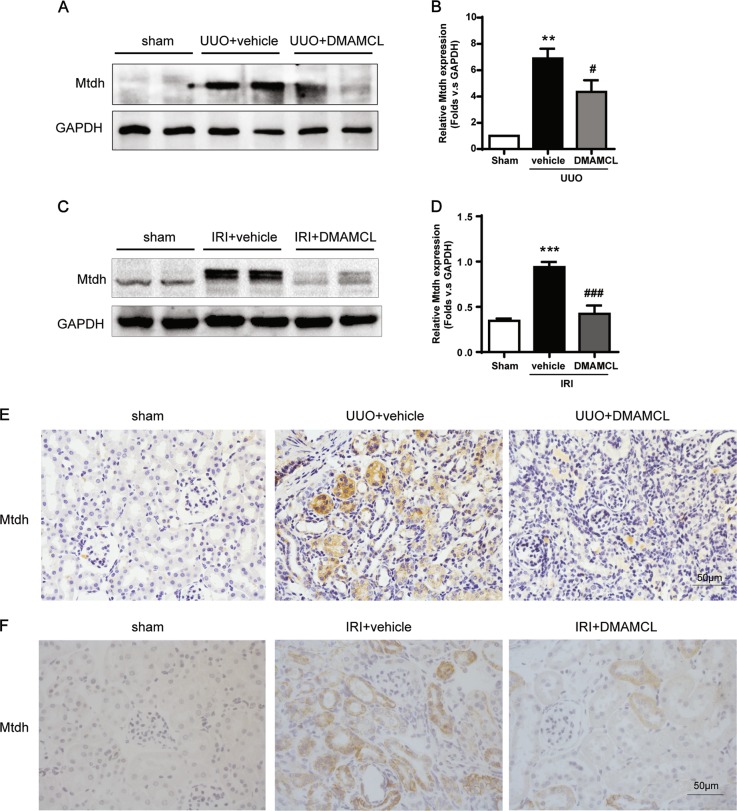
DMAMCL/MCL Inhibited Mtdh Expression in the UUO and IRI mice. **a** Representative Western blot showing Mtdh levels in the UUO mice. **b** Relative levels of the Mtdh protein in the blot shown in (**a**). **P < 0.01 compared with the sham group; #*P* < 0.05 compared with the vehicle group. **c** Representative Western blot showing Mtdh levels in the IRI mice. **d** Relative levels of the Mtdh protein in the blot shown in (**c**). ***P < 0.001 compared with the sham group; ###*P* < 0.001 compared with the vehicle group. **e** Representative Mtdh IHC staining in the UUO mice. **f** Representative Mtdh IHC staining in the IRI mice. Scale bar in (**e**) and (**f**) represents 50 μm. All the data are presented as the mean ± SEM of at least three independent experiments

To elucidate the underlying mechanism by which DMAMCL functioned in Renal fibrosis, Mtdh was investigated. As shown in Fig. 4, Mtdh was markedly upregulated in both the UUO model and IRI models, while DMAMCL reduced its expression. These results suggest that Mtdh might have a pathogenic role in the initiation or development of fibrosis.

**Fig. 5:**
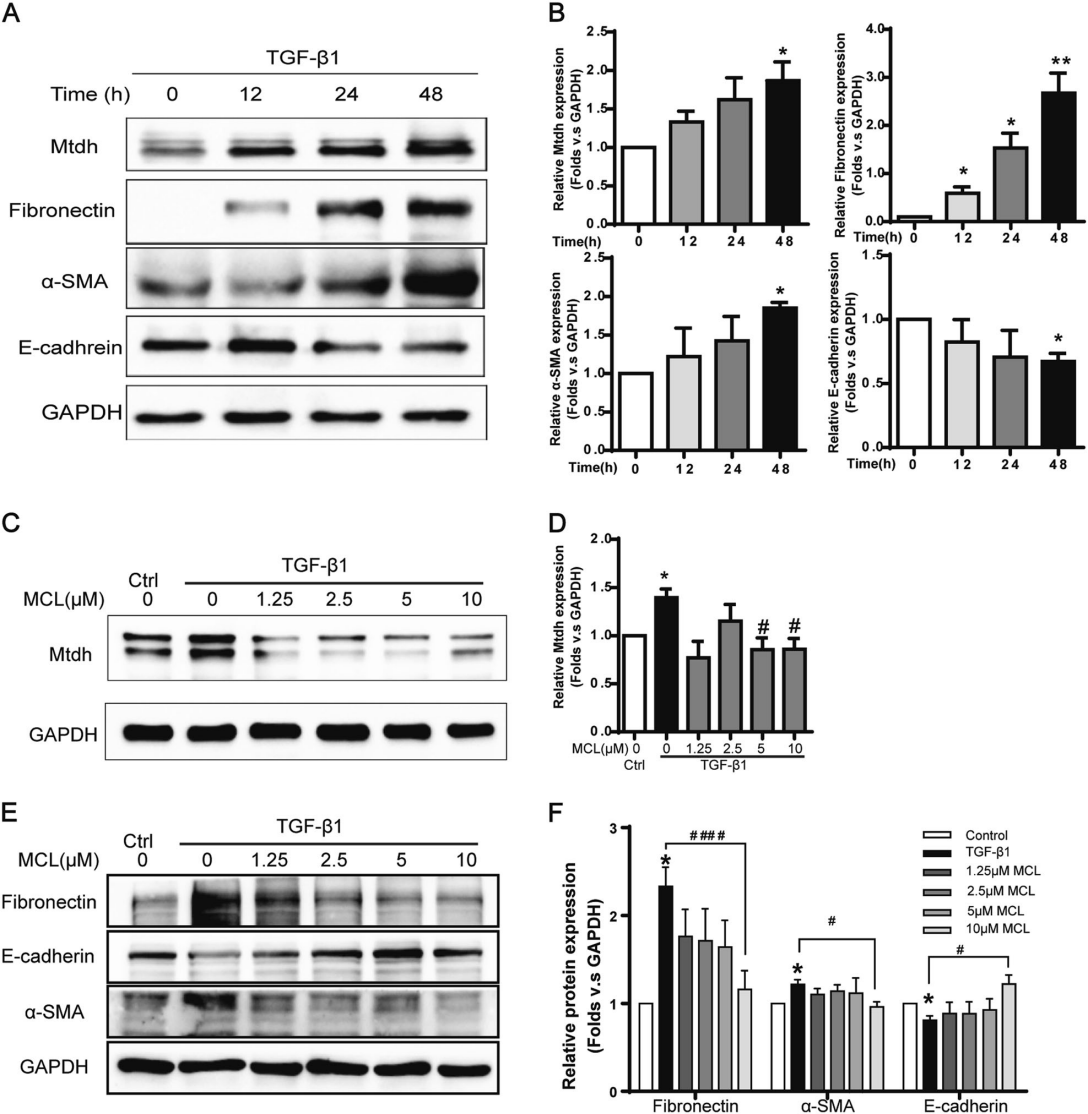
DMAMCL/MCL Inhibited both Mtdh and EMT in vitro. **a** Representative Western blot shows Mtdh and EMT markers (fibronectin, α-SMA, and E-cadherin) in mTECs cell lysates treated with TGF-β1 (5 ng/ml) for 0, 12, 24, 48 h. **b** Relative levels of the Mtdh and EMT marker proteins compared to GAPDH. *P < 0.05 compared with 0 h; **P < 0.01 compared with 0 h. **c** mTECs were coincubated with MCL (0, 1.25, 2.5, 5, or 10 μM) and TGF-β1 (5 ng/ml). Cells were harvested 48 h later, and cell lysates were immunoblotted to detect Mtdh expression. **d** Relative levels of the Mtdh, proteins compared to GAPDH. *P < 0.05 compared with the control group; #*P* < 0.05 compared with TGF-β1 group. **e** Representative Western blot on EMT markers in MCL- TGF-β1 coincubation cell lysates samples. **f** Relative levels of the EMT markers in the blot shown in (**e**). *P < 0.05 compared with the control group; #*P* < 0.05 compared with the TGF-β1 group; ####*P* < 0.0001 compared with the TGF-β1 group. The data are presented as the mean ± SEM of at least three independent experiments

Intriguingly, Mtdh expression was increased in a TGF-β1-induced fibrosis cellular model in mTECs in a time dependent manner (Fig. 5a, b). With the addition of MCL, the active form of DMAMCL, in this model, Mtdh expression was significantly suppressed (Fig. 5c, d), and EMT markers were relieved (Fig. 5e, f) compared with TGF-β1 group. MCL showed efficacy in partly reversing EMT in vitro, and Mtdh might be one of its mechanism.

### Mtdh might be a novel mediator promoting kidney fibrotic change

**Fig. 6:**
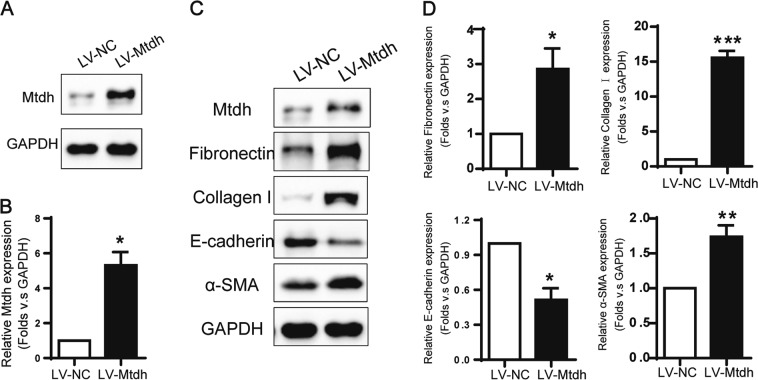
Mtdh Promotes EMT in mTECs. **a** Representative Western blot showing Mtdh levels in LV-Mtdh and LV-NC cells. **b** Relative levels of Mtdh in these two groups. *P < 0.05 compared with LV-NC. **c** Representative Western blots showing levels of EMT markers in LV-Mtdh and LV-NC cells. **d** Relative protein expression shown in (**c**). *P < 0.05 compared with LV-NC; **P < 0.01 compared with LV-NC; ***P < 0.001 compared with LV-NC. The data are presented as the mean ± SEM of at least three independent experiments

As mentioned previously, we found a relationship between Mtdh expression and DMAMCL/MCL treatment in two mouse renal fibrosis models, and Mtdh might be involved in the pro-fibrotic process. To further explore the underlying mechanism of Mtdh in EMT, we established a stable Mtdh overexpression mTEC cell line (LV-Mtdh) and used an empty vector to generate a negative control cell line (LV-NC). After screening positive stable cell lines with 10 ng/ml puromycin, the Mtdh level was significantly increased in LV-Mtdh cell lysates compared with LV-NC lysates (Fig. 6a). We detected the EMT phenotype in these two cell lines. As shown in Fig. 6, fibronectin, α-SMA and collagen I expression was upregulated in the LV-Mtdh group compared with the LV-NC group. E-cadherin was expressed at a lower level in the LV-Mtdh group than in the LV-NC group. Based on these trends, Mtdh might have a pathogenic role in fibrogenesis under basal conditions (without TGF-β1 stimulation).

**Fig. 7:**
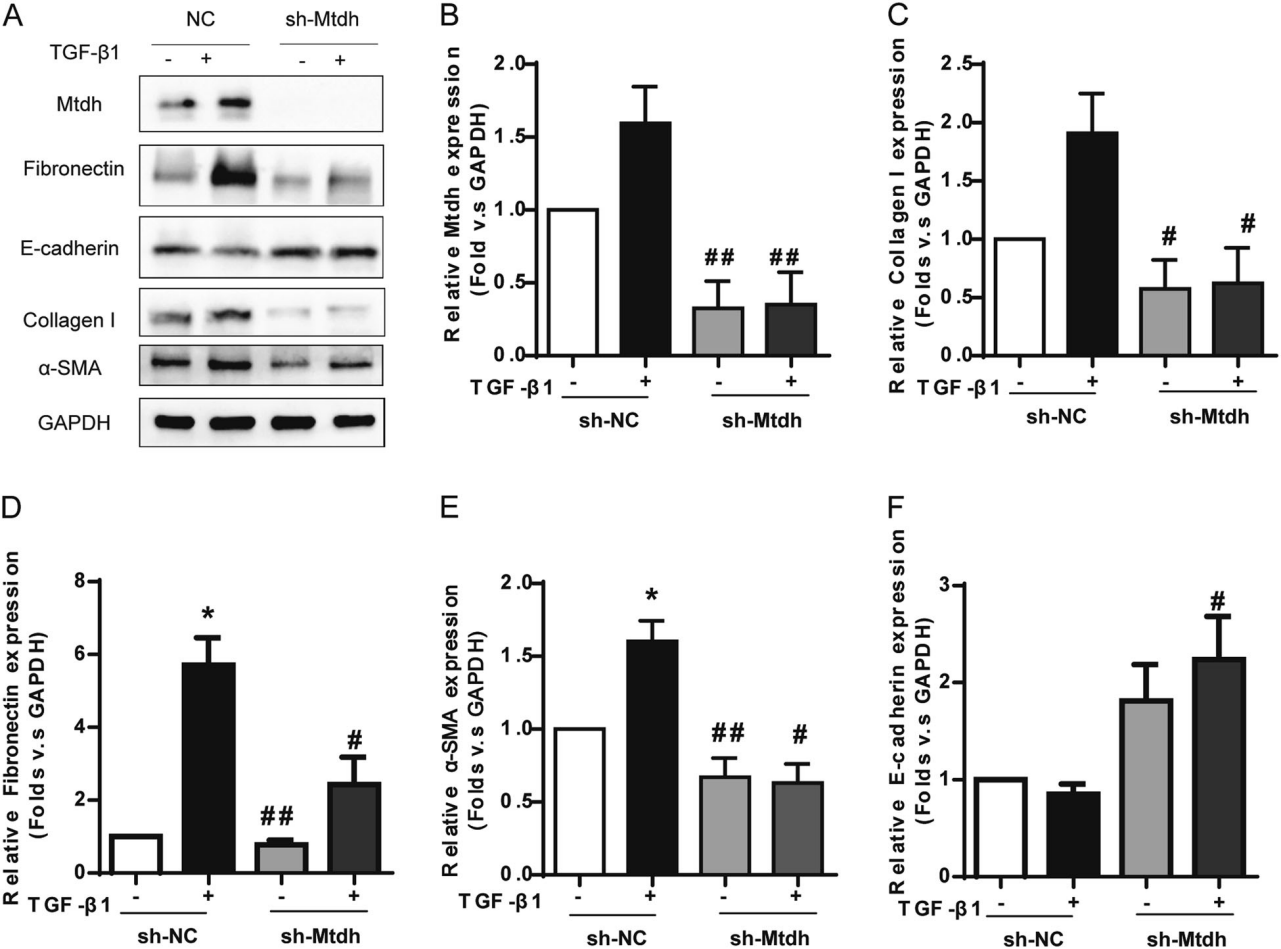
Knockdown of Mtdh Relieved the TGF-β1-induced EMT in mTECs. **a** Representative Western blot analyses of sh-NC and sh-Mtdh cells. **b**, **c**, **d**, **e** and **f** Relative protein expression shown in (**a**). *P < 0.05 compared with sh-NC; #*P* < 0.05 compared with sh-NC + TGF-β1; ##*P* < 0.01 compared with sh-NC + TGF-β1. The data are presented as the mean ± SEM of at least three independent experiments

Mtdh stable-knockdown cell lines were constructed (sh-Mtdh) as previously described, and sh-NC served as a negative control to further evaluate the effect of Mtdh on renal fibrosis. Dramatic decreases in fibronectin, collagen I and α-SMA expression were observed in the sh-Mtdh group, even in the presence of TGF-β1 stimulation. Consistent with the levels of mesenchymal cell markers, the expression of the epithelial cell marker E-cadherin was increased after Mtdh knockdown (Fig. 7).

### MCL relieves the fibrotic phenotype induced by Mtdh overexpression

**Fig. 8:**
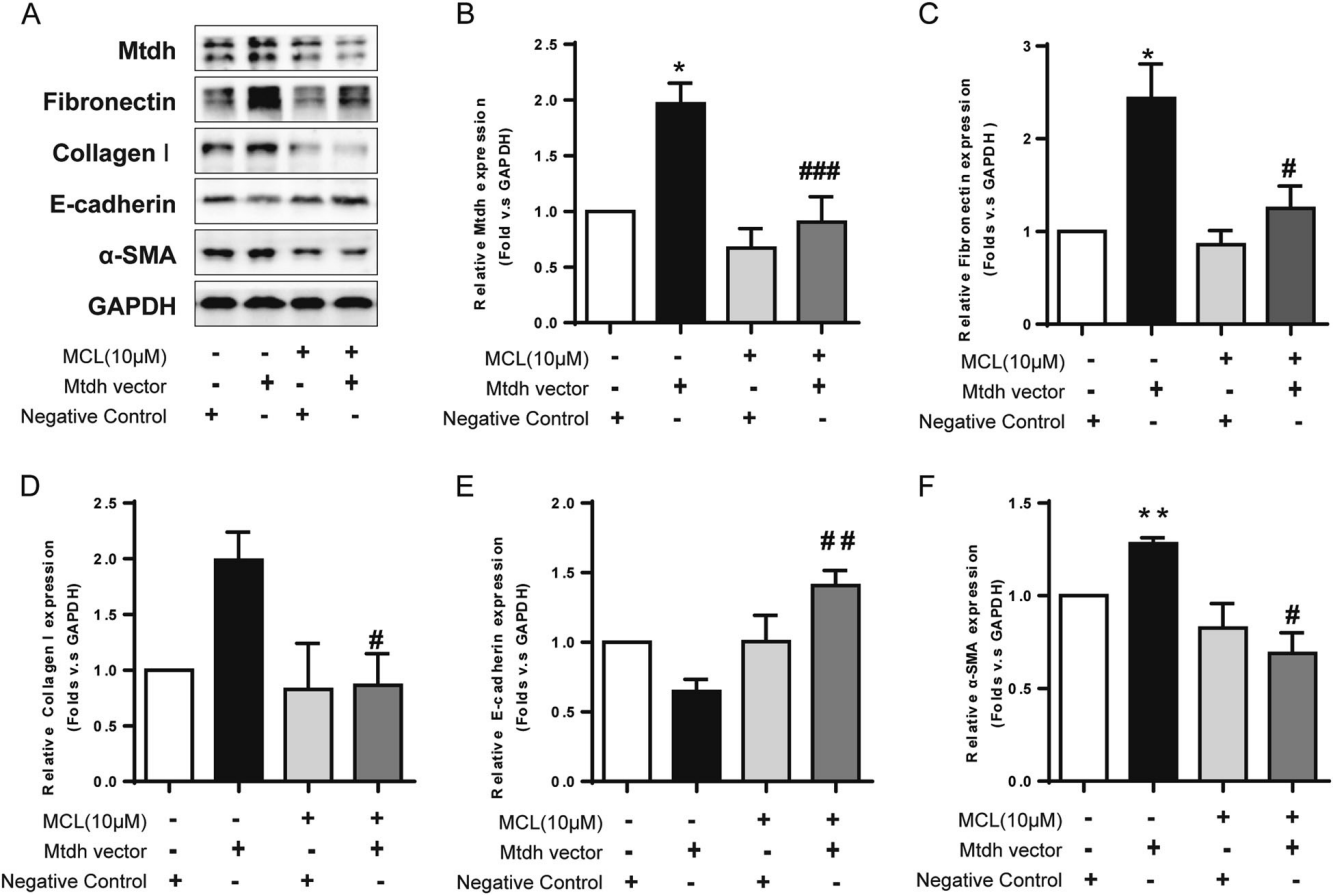
MCL Relieves the Fibrotic Phenotype Induced by Mtdh Overexpression. **a** Representative Western blot analyses of levels of Mtdh and EMT markers. **b**, **c**, **d**, **e** and **f** Relative protein expression shown in (**a**). *P < 0.05 compared with the NC group; **P < 0.01 compared with the NC group; #*P* < 0.05 compared with the Mtdh vector group; ##*P* < 0.01 compared with the Mtdh vector group; ###*P* < 0.001 compared with the Mtdh vector group; #### *P* < 0.0001 compared with the Mtdh vector group. The data are presented as the mean ± SEM of at least three independent experiments

Because Mtdh plays a pivotal role in renal fibrosis, and DMAMCL/MCL inhibits its expression and exerts anti-fibrotic effects, we hypothesized that the therapeutic effects of DMAMCL/MCL might be attributed to the mechanism by which Mtdh expression is inhibited. mTECs transiently transfected with the Mtdh target plasmid were treated with MCL in vitro for 24 h to verify this hypothesis. As shown in Fig. 8, Mtdh expression was increased in the transfection group, and MCL significantly inhibited its expression. The expression of the fibrotic marker fibronectin, α-SMA, and collagen I was decreased, while their levels were increased in cells overexpressing Mtdh. E-cadherin exhibited the opposite trends. Thus, MCL therapy partially reverses the EMT driven by Mtdh overexpression. In other words, MCL relieves the EMT in renal TECs by suppressing Mtdh expression.

### Mtdh selectively modulates BMP/Smad1/5/9 signalling rather than TGF-β/Smad2/3 signalling

**Fig. 9:**
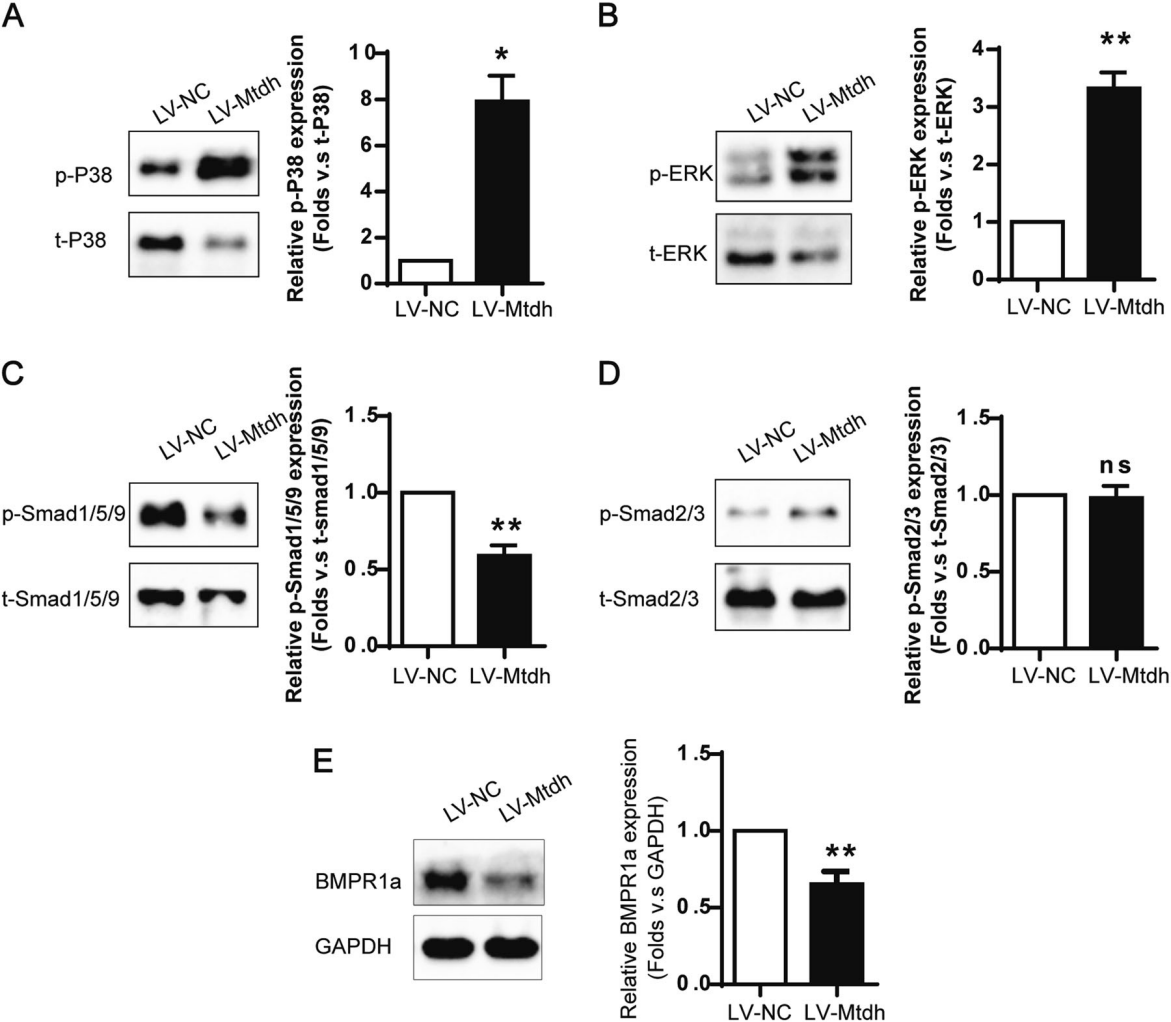
Mtdh Activates BMP/MAPK Signalling. **a** Representative Western blot analyses of phosphorylated P38 MAPK. *P < 0.05 compared with LV-NC. **b** Representative Western blot analyses of phosphorylated ERK. **P < 0.01 compared withLV-NC. **c** Representative Western blot analyses of phosphorylated Smad1/5/9. **P < 0.01 compared with LV-NC. **d** Representative Western blot analyses of phosphorylated Smad2/3. ns indicates *P* > 0.05 compared with LV-NC. **e** Representative Western blot analyses of BMPR1A. **P < 0.01 compared with LV-NC. The data are presented as the mean ± SEM of at least three independent experiments

We next examined whether Mtdh was involved in crucial modulatory pathways, such as the TGF-β and BMP signalling. The BMP/BMPR1A/Smad1/5/9 pathway competitively antagonizes TGF-β/Smad2/3 signalling, and both p-Smad1/5/9 and p-Smad2/3 can combine with Smad4 to form a complex for transport into the nucleus, where this complex functions to regulate gene transcription. As shown in Fig. 9, Mtdh overexpression decreased the levels of BMPR1A and phosphorylated Smad1/5/9 in the absence of TGF-β1 stimulation. However, no changes in levels of Smad2/3 phosphorylation were observed in cells transfected with LV-Mtdh and LV-vector.

The MAPK pathway, which includes P38 MAPK and ERK, is a non-classical BMP signalling pathway that does not involve Smads and plays an important role in many aspects of fibrogenesis. Here, Mtdh overexpression increased P38 MAPK and ERK phosphorylation. As shown in Fig. 9, significant increases in P38 MAPK and ERK phosphorylation were observed compared with total P38 MAPK and ERK in the LV-Mtdh group.

**Fig. 10:**
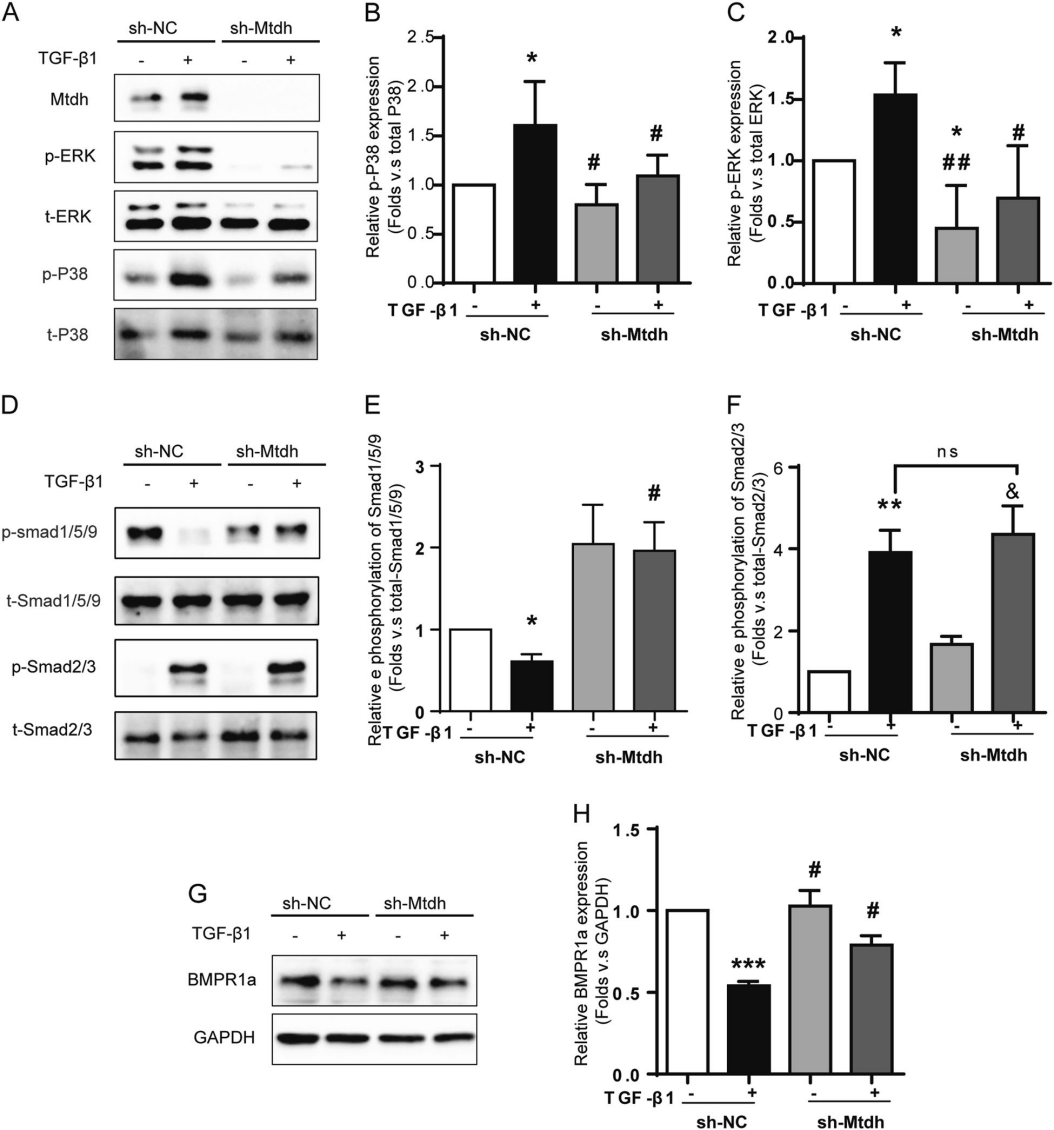
Mtdh knockdown inhibits TGF-β1-induced BMP/MAPK signalling in vitro. **a** Representative Western blot analyses of MAPK signalling intermediates in sh-NC and sh-Mtdh cells. **b**, **c** Relative protein expression shown in (**a**). *P < 0.05 compared with sh-NC; #*P* < 0.05 compared with sh-NC + TGF-β1; ##*P* < 0.01 compared with sh-NC + TGF-β1. **d** Representative Western blot analyses of Smad1/5/9 and Smad2/3 phosphorylation in sh-NC and sh-Mtdh cells. **e**, **f** Relative protein expression shown in (**d**). *P < 0.05 compared with sh-NC; ** *P* < 0.01 compared with sh-NC; #*P* < 0.05 compared with sh-NC + TGF-β1; & *P* < 0.05 compared with sh-Mtdh; ns *P* > 0.05 compared with sh-NC + TGF-β1. **g** Representative Western blot analyses of BMPR1a in sh-NC and sh-Mtdh cells. **h** Relative protein expression shown in (**g**). ***P < 0.0001 compared with sh-NC; #*P* < 0.05 compared with sh-NC + TGF-β1. The data are presented as the mean ± SEM of at least three independent experiments

The same experiment was performed in Mtdh knockdown cells with or without TGF-β1 treatment to confirm the effects of Mtdh on TGF-β, BMP and MAPK signalling. As shown in Fig. 10, consistent with the conclusions listed above, sh-Mtdh reduced TGF-β1-induced Smad1/5/9 phosphorylation, while Smad2/3 phosphorylation was not affected. Also, sh-Mtdh decreased phosphorylation level of P38 MAPK and ERK. Based on these results, Mtdh might act upstream of the P38 MAPK/ERK pathway and regulate the phosphorylation of these proteins under fibrotic conditions.

## Discussion

In the present study, we provide evidence of the effects of DMAMCL/MCL, derivatives of PTL, on alleviating renal fibrosis in vivo and in vitro. Additionally, a novel pro-fibrotic mediator was identified—Mtdh. Coincidentally, DMAMCL/MCL suppressed Mtdh expression in renal fibrotic models in vivo and in vitro. We provide strong evidence that DMAMCL specifically reduced the EMT induced by suppressing Mtdh expression in vitro. Therefore, this study identified a novel pro-fibrotic mediator, Mtdh, and was the first study to reveal the anti-fibrotic potential of DMAMCL/MCL in treating renal diseases by suppressing the Mtdh/BMP/MAPK axis.

DMAMCL/MCL, isolates of *Michelia*, present promising anti-fibrotic efficacy. These compounds are derived from PTL, an anti-inflammatory drug with a long history of use in treating various diseases. With improved water solubility and stability, DMAMCL/MCL are expected to have more extensive applications. MCL has been shown to inhibit glioma cell growth by inducing apoptosis in colitis-associated cancer cells [2], activate pyruvate kinase M2 (PKM2) to suppresses leukaemia [1], suppress LPS-induced neuroinflammatory responses [3], and alleviate liver steatosis in diabetic mice by reducing inflammation and amplifying autophagy [8]. This study is the first to verify the therapeutic effect of MCL on renal fibrosis and its underlying mechanism. The MCL intervention attenuated the fibrotic morphological changes observed in Masson’s trichrome staining and levels of ECM proteins in established UUO mice and IRI mice. DMAMCL could further protect the kidney, as serum creatine and urea levels were decreased after treatment.

MCL may reverse fibrosis through many pathways; here, we focused on Mtdh, a novel factor involved in kidney fibrogenesis, as described above. Based on the results from both in vivo and in vitro studies, DMAMCL/MCL suppressed Mtdh expression. Specifically, MCL suppressed EMT in mTECs induced by Mtdh overexpression. Thus, Mtdh might be a selective target of DMAMCL/MCL that mediates the anti-fibrotic effect. However, we were unable to clarify the correlation between the Mtdh level and the severity of fibrosis due to insufficient samples.

Certain previous studies have focused on the role of Mtdh in the EMT. A positive correlation between Mtdh overexpression and EMT in cancers has been reported [3]. However, little evidence of Mtdh upregulation in kidney diseases is available. According to Peng et al., Mtdh is involved in TGF-β1-induced EMT by modulating P38 MAPK phosphorylation in the HK-2 human kidney tubular epithelial cell line [30]. Wei et al. conducted a similar study on HG-induced EMT in HK-2 cells and found that Mtdh is also involved in this non-canonical EMT process, possibly by activating Rho kinase [31]. However, those studies focused only on the cellular aspect, and their evidence was not very strong. In our study, we first designed a time course experiment to examine Mtdh expression in mTECs following TGF-β1 stimulation and found that Mtdh expression was significantly increased as early as 12 h and reached its plateau at 24 h, as no difference was observed between 24 and 48 h. We also showed for the first time that Mtdh expression was upregulated in fibrotic animal models. This study was also the first to examine the effects of Mtdh modulation on the BMP pathway.

EMT has been studied for the past decade to gain a better understanding of its roles in tumourigenesis and organ fibrosis. Intrinsic cells in the kidney undergo EMT during injury, which is an important mechanism in renal fibrosis [32]. At day 14, the obstructed kidney showed typical fibrotic characteristics, such as inflammatory cell infiltration, tubular degeneration and atrophy, expansion of the interstitium and ECM accumulation. The morphological changes were consistent with the upregulation of fibronectin and α-SMA expression, as well as the reduction in E-cadherin expression. Based on these findings, EMT is present in the UUO model of renal fibrosis. Because Mtdh has been shown to participate in EMT process in tumours and the EMT in renal TECs might also be an important source of myofibroblasts, we examined Mtdh expression and found substantially higher levels of Mtdh in UUO mice than in sham mice. The similar results were showed in IRI model that Mtdh is upregulated in the IRI condition. Therefore, we hypothesize that Mtdh might promote EMT during renal fibrogenesis.

We constructed two stable mTEC lines to clarify whether Mtdh exacerbates EMT in vitro; one line stably overexpressed Mtdh (LV-Mtdh, LV-NC as control), and the other was a stable Mtdh-knockdown line (sh-Mtdh, sh-NC as control). Compared with the LV-NC group, the LV-Mtdh group showed a higher EMT potential. Fibronectin, collagen I, and α-SMA were significantly upregulated in the LV-Mtdh group. Mtdh-knockdown cells presented a low sensitivity to TGF-β1. Compared with the sh-NC group, the sh-Mtdh group did not readily acquire the EMT phenotype upon TGF-β1 stimulation. These in vitro results provided further support for our hypothesis that Mtdh is involved in the promotion of kidney fibrosis. The effect of Mtdh on animals requires further investigation.

TGF-β/Smads and BMP/Smads are antagonists of the pathogenesis of renal fibrosis. TGF-β1 produces its downstream effects by binding to TβRII and subsequently activating TβRI, leading to Smad2/3 phosphorylation and the recruitment of Smad4 to form the R-Smad complex that translocates into the nucleus to regulate the transcription of pro-fibrotic genes [33, 34]. BMP is another extracellular signal involved in fibrogenesis. Similarly, BMP-7 binds to BMPR1a (also known as Alk3) and activates this signalling pathway by phosphorylating Smad1/5/9 and competitively binding to Smad4, resulting in reduced ECM accumulation [35]. Moreover, BMP-7 reduces Smad3 binding to DNA by increasing SnoN expression, without interfering with Smad3 phosphorylation or degradation [36].

As shown in the present study, Mtdh overexpression reduced BMP7 and BMPR1a levels and increased the levels of phosphorylated Smad1/5/9, but not Smad2/3, suggesting that Mtdh might function upstream of BMP/Smad1/5/9 rather than TGF-β/Smad2/3. The data obtained from Mtdh knockdown cells were consistent with these observations. The P38 MAPK and ERK pathway, a non-canonical BMP signalling pathway, were associated with Mtdh. According to our data, Mtdh increased the phosphorylation of P38 MAPK and ERK. To the best of our knowledge, this study is the first to verify Mtdh expression in the UUO mice and IRI mice and to link Mtdh and the BMP pathway.

In conclusion, we identified Mtdh as a novel modulator of fibrogenesis. Mtdh regulates BMP/MAPK signalling and mainly influences Smad1/5/9 phosphorylation, instead of Smad2/3 phosphorylation, suggesting that Mtdh exerts a selective effect on BMP signalling rather than the TGF-β/Smad2/3 pathway. The administration of the DMAMCL/MCL intervention to UUO mice and TGF-β1-treated mTECs exerts a potent anti-fibrotic effect by suppressing Mtdh expression. This study provides a promising lead compound for developing an effective anti-fibrotic therapy.
