# Correction: Micheliolide ameliorates renal fibrosis by suppressing the Mtdh/BMP/MAPK pathway

**DOI:** 10.1038/s41374-019-0301-2

**Published:** 2019-08-16

**Authors:** Fenfen Peng, Hongyu Li, Shuting Li, Yuxian Wang, Wenting Liu, Wangqiu Gong, Bohui Yin, Sijia Chen, Ying Zhang, Congwei Luo, Weidong Zhou, Yihua Chen, Peilin Li, Qianyin Huang, Zhaozhong Xu, Haibo Long

**Affiliations:** 1 Department of Nephrology, Zhujiang Hospital, Southern Medical University, 510280 Guangzhou, China; 2 Department of Gerontology, ZhuJiang Hospital, Southern Medical University, Guangzhou, 510280 China; 3 Department of Nephrology, The First Hospital of Changsha, Changsha, 410000 China; 4 Department of Nephrology, The Second Affiliated Hospital, Guangzhou Medical University, Guangzhou, 510260 China

**Keywords:** Drug regulation, End-stage renal disease

**Correction to: Laboratory Investigation**

10.1038/s41374-019-0245-6

Published online 11 April 2019

The authors would like to apologize for the following errors in this paper:

The word “creactine” should be “creatinine” in Fig. 3c.

**Fig. 3:**
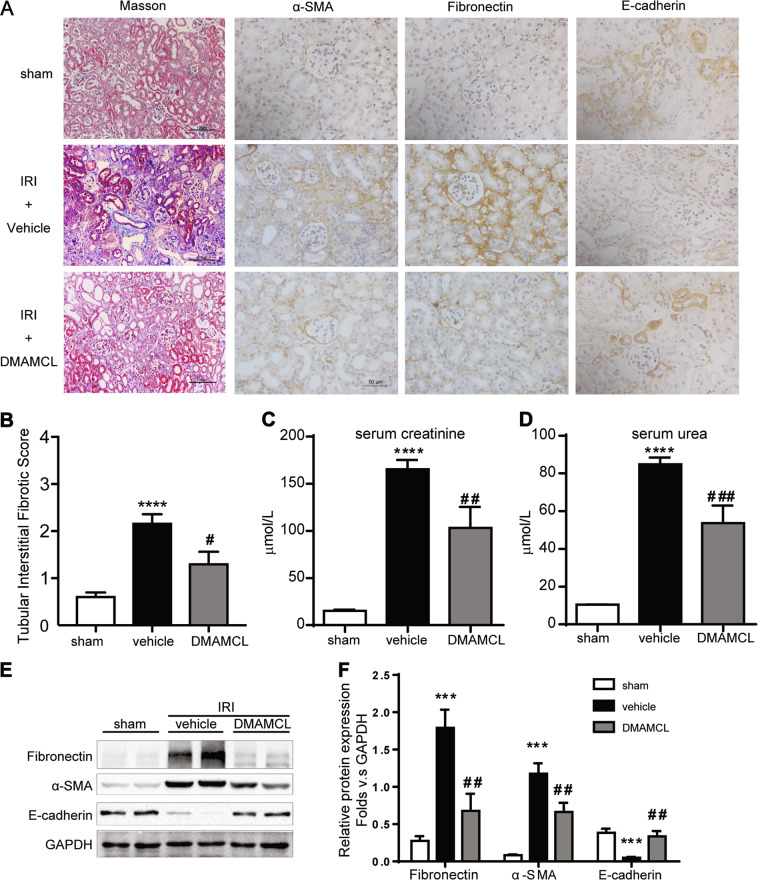
DMAMCL protects kidney from fibrosis in the IRI mice. **a** Representative micrographs of Masson's trichrome staining and IHC staining for α-SMA, fibronectin, and E-cadherin in the injured kidneys. Scale bar in Masson’s trichrome staining is 100 μm; in IHC staining is 50 μm. **b** Quantification of renal tubular interstitial fibrotic score. ****P < 0.0001 compared with the sham group; ^#^*P* < 0.05 compared with the vehicle group. **c** Serum creatine level in the IRI mice. ****P < 0.0001 compared with the sham group; ^##^*P* < 0.01 compared with the vehicle group. **d** Serum urea level in the IRI mice. ****P < 0.0001 compared with the sham group; ^###^*P* < 0.001 compared with the vehicle group. **e** Representative bands from Western blot analyses of the levels of the α-SMA, fibronectin, and E-cadherin proteins in kidney tissues from the IRI mice. **f** Relative protein levels as determined by the Western blot assay **e**. ***P < 0.001 compared with the sham group; ^##^*P* < 0.01 compared with the vehicle group. n=6 mice per group, all the data are presented as the mean ± SEM of at least three independent experiments

The term “mg/dl” should be “µmol/L” in Fig. 3c, d.

The first acknowledgement should read, “This work was supported by the National Natural Science Foundation of China (No. 81673792, 81704134, 81600624, 81800612, U1801288, and 81873346)…”

The address for Zhaozhong Xu should be Department of Nephrology, Zhujiang Hospital, Southern Medical University, 510280 Guangzhou, China.

